# Physicochemical and Computational Study of the Encapsulation of Resv-4′-LA and Resv-4′-DHA Lipophenols by Natural and HP-β-CDs

**DOI:** 10.3390/ijms26157454

**Published:** 2025-08-01

**Authors:** Ana Belén Hernández-Heredia, Dennis Alexander Silva-Cullishpuma, José Pedro Cerón-Carrasco, Ángel Gil-Izquierdo, Jordan Lehoux, Léo Faion, Céline Crauste, Thierry Durand, José Antonio Gabaldón, Estrella Núñez-Delicado

**Affiliations:** 1 Molecular Recognition and Encapsulation Research Group (REM), Health Sciences Department, Universidad Católica de Murcia (UCAM), Campus de los Jerónimos 135, E-30107 Guadalupe, Spain; abhernandez@ucam.edu (A.B.H.-H.); dasilva@ucam.edu (D.A.S.-C.); 2 Centro Universitario de la Defensa, Universidad Politécnica de Cartagena, C/Coronel López Peña s/n, Base Aérea de San Javier, E-30720 Santiago de la Ribera, Murcia, Spain; jose.ceron@cud.upct.es; 3 Research Group on Quality, Safety, and Bioactivity of Plant-Derived Foods, Department of Food Science and Technology, Centro de Edafologia y Biologia Aplicada del Segura-Consejo Superior de Investigaciones Científicas (CEBAS-CSIC,) Campus de Espinardo-25, E-30100 Murcia, Spain; angelgil@cebas.csic.es; 4 Institut des Biomolécules Max Mousseron (IBMM), Pôle Chimie Balard Recherche, UMR 5247-CNRS, Faculty of Pharmacy, Université de Montpellier-École Nationale Supérieure de Chimie de Montpellier (ENSCM), 34000 Montpellier, France; jordan.lehoux@umontpellier.fr (J.L.); leo.faion@umontpellier.fr (L.F.); celine.crauste@umontpellier.fr (C.C.); thierry.durand@umontpellier.fr (T.D.)

**Keywords:** lipophenols, fatty acids, cyclodextrins, bioactive compounds, micelles, nanoencapsulation

## Abstract

This study investigates the self-assembly and host–guest complexation behaviour of novel resveratrol-based lipophenols (LipoResv)—resveratrol-4′-linoleate (Resv-4′-LA) and resveratrol-4′-docosahexaenoate (Resv-4′-DHA)—with hydroxypropyl-β-cyclodextrins (HP-β-CDs). These amphiphilic molecules display surfactant-like properties, forming micellar aggregates in aqueous media. Fluorescence spectroscopy was used to determine the critical micelle concentration (CMC), revealing that LipoResv exhibit significantly lower CMC values than their free fatty acids, indicating higher hydrophobicity. The formation of inclusion complexes with HP-β-CDs was evaluated based on changes in CMC values and further confirmed by dynamic light scattering (DLS) and molecular modelling analyses. Resv-4′-LA formed 1:1 complexes (Kc = 720 M^−1^), while Resv-4′-DHA demonstrated a 1:2 stoichiometry with lower affinity constants (K_1_ = 17 M^−1^, K_2_ = 0.18 M^−1^). Environmental parameters (pH, temperature, and ionic strength) significantly modulated CMC and binding constants. Computational docking and molecular dynamics simulations supported the experimental findings by revealing the key structural determinants of the host–guest affinity and micelle stabilization. Ligand efficiency (LE) analysis further aligned with the experimental data, favouring the unmodified fatty acids. These results highlight the versatile encapsulation capacity of HP-β-CDs for bioactive amphiphile molecules and support their potential applications in drug delivery and functional food systems.

## 1. Introduction

Polyphenols and essential omega-3-polyunsaturated fatty acid (n-3 PUFA) have been widely recognized as molecules with premium beneficial effects on health [1,2]. On the other hand, chemical bonding between the two structures leading to n-3 lipophenol derivatives (or phenolipids) has been studied in a growing number of works over the last decade. Some examples of lipophenol-related studies highlighted their presence in food or vegetable matrices, while others have reported a synthetic lipophenol developed for therapeutic application [3]. They have been designed to link in the same molecule, to share the potential health benefits of both the polyphenol and the fatty acid, and at the same time, increase the hydrophobicity of weak hydrophobic polyphenol for a better cell penetration, and the protection of oxidation in lipid membrane or lipid rich tissue [4]. In this context, we have previously synthesized combinations of resveratrol with linoleic or docosahexaenoic acids, resveratrol-4′-linoleate (Resv-4′-LA), and resveratrol-4′-docosahexaenoate (Resv-4′-DHA), respectively, for testing their activity to reduce oxidative stress in retinal pigment epithelial cells for age-related macular degeneration purposes [5]. In addition, in in vitro assays, (Resv-4′-LA) exhibited a drastic reduction in the exacerbated pathological endothelial permeability by inhibiting the activity and expression of MMP-9 metalloproteases and, consequently, preserving the endothelial intercellular junction integrity [6], thus being a promising group contributing to the absence of pathological events in populations that are already at risk and predisposed to occur (primary prevention) or improving the ischemic damage with already established pathological events of a relatively reduced or controlled extent (secondary prevention).

Despite the plausible potential effects of this compound, the accentuated hydrophobic nature, the difficulty of obtaining it by chemical or enzymatic synthesis with good yields, the scarcity of work related to their mapping in plants, as well as in vitro and in vivo assays that shed some light on their bioavailability, absorption, and metabolism, we recommend urgently addressing this task, attempting to solve low aqueous solubility by using cyclodextrins (CDs) for later preclinical assays.

**Figure 1:**
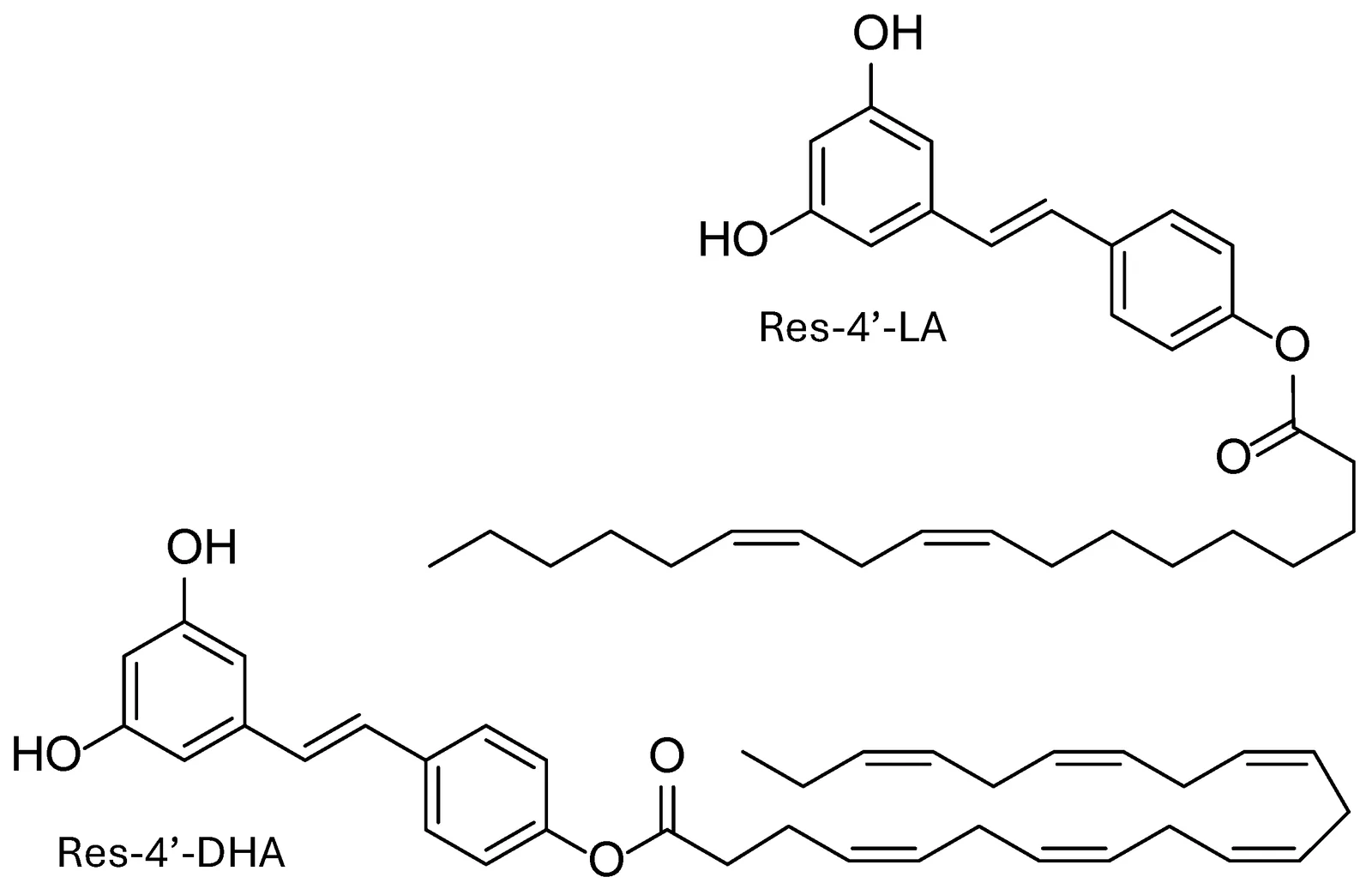
Structure of resveratrol lipophenols (LipoResv): resveratrol-4′-linoleate (Resv-4′-LA) and resveratrol-4′-docosahexaenoate (Resv-4′-DHA).

The solubilization of bioactive compounds remains a significant challenge in food, pharmaceutical, and biomedical research. In fact, resveratrol lipophenols or resveratrol phenolipids (LipoResv) are amphiphilic molecules composed of some of a polar head group (resveratrol) and a hydrophobic aliphatic chain derived from fatty acids (Figure 1). In aqueous environments, the hydrophobic segments tend to associate through hydrophobic interactions, resulting in the formation of a non-polar core that excludes water molecules. Concurrently, the polar head groups orient towards the surrounding aqueous phase.

This amphiphilic nature drives the spontaneous self-assembly of LipoResv into organized supramolecular structures, analogous to those formed by classical lipids [7].

Micellar systems are commonly employed as supramolecular hosts to enhance the solubilization of different poorly water-soluble compounds [8]. The ability of lipophenols to self-assemble into micelle-like aggregates imparts a dual functionality, enabling them to act both as host molecules and as structural components of micellar assemblies. This dual role introduces additional complexity to the equilibria in the solution, as their behaviour is highly dependent on the surrounding environment [9].

Like micellar systems, CDs have been widely used for the solubilization of poorly water-soluble drugs and other hydrophobic compounds [10,11]. CDs are cyclic oligosaccharides with a hydrophobic cavity, enabling the formation of dynamic inclusion complexes with hydrophobic molecules [12]. β-CDs are the most accessible and widely used native type, while numerous derivatives with modified properties have been synthesized. Inclusion in CDs alters the physicochemical properties of guest molecules, enhancing the solubility, stability, and controlled release, among other effects. This versatility makes CDs valuable in diverse fields, including food, pharmaceuticals, cosmetics, and environmental applications [10,13,14,15]. For example, their use in water decontamination has been studied in recent years, linked both to the removal of dies derived from the textile industry [16,17], and to the removal of persistent emerging contaminants in wastewater [18,19,20]. Moreover, their use has also been described for the controlled release of agrochemicals, such as chlorpyrifos [21].

It is important to note that the complexation ability of CDs is influenced not only by the specific type of CDs employed, but also by the physicochemical characteristics of the guest molecule [11]. In this sense, the efficiency and stability of complex formation can vary depending on the compatibility between the structural and chemical properties of the host and guest [22]. Furthermore, multiple modes of inclusion are possible [10]. Unlike micellar systems, CDs are structurally well-defined and do not rely on aggregation equilibria for their functionality. Nevertheless, their inclusion capacity and interaction with guest molecules can be significantly modulated by external conditions such as temperature, pH, and ionic strength [23].

The critical micelle concentration (CMC) is a fundamental characteristic of surfactants, representing the concentration at which self-assembly into micelles begins. Due to their amphiphilic nature, lipophenols exhibit surfactant-like behaviour [24]. The formation of micelles induces measurable changes in various physicochemical properties of the solution, providing each one with an analytical signal that can be measured and transduced to obtain a parametric value by applying a chemical measurement process. Consequently, several analytical techniques such as electrical conductivity, surface tension measurements, nuclear magnetic resonance (NMR) spectroscopy, UV-Vis absorption, or fluorescence spectroscopy, between others, can be employed to determine the CMC [12,23,24,25,26,27].

It is important to recognize that not all analytical methodologies are universally applicable to every compound; the selection of an appropriate technique must be guided by the specific physicochemical behaviour of the compound under study [28]. The wide range of empirical approaches available for determining the CMC likely reflects the inherent complexity of the micellization process. Although there are advanced models and simulations that describe micelle formation in detail, these are often not designed to analyze experimental data directly. As a result, they usually do not include equations where the CMC can be used as a fitting parameter.

The use of a fluorometric method for determining the CMC is motivated, in part, by their higher sensitivity and accuracy, particularly for compounds with low CMC values [26]. This approach offers advantages over other techniques that may be less effective, due to technical limitations or incompatibility with the physicochemical properties of certain compounds [29].

This is the first study to analyze the complexation between CDs and LipoResv. In this work, we studied the structural and aggregation behaviour of LipoResv, specifically Resv-4′-LA and Resv-4′-DHA, in the presence of CDs. Moreover, the effect of hydroxypropyl-β-cyclodextrins′ (HP-β-CDs) concentration, temperature, pH, and ionic strength in the complexation process will be evaluated. To elucidate the aggregation behaviour of LipoResv and to investigate the mechanism of complexation with HP-β-CDs across different environmental conditions, fluorescence spectroscopy was employed to determine the CMC, while dynamic light scattering (DLS) was used to assess particle size and aggregation profiles. These experimental findings are further supported by computational models, providing insight into the molecular interactions underlying the complexation process.

## 2. Results and Discussion

**Figure 2:**
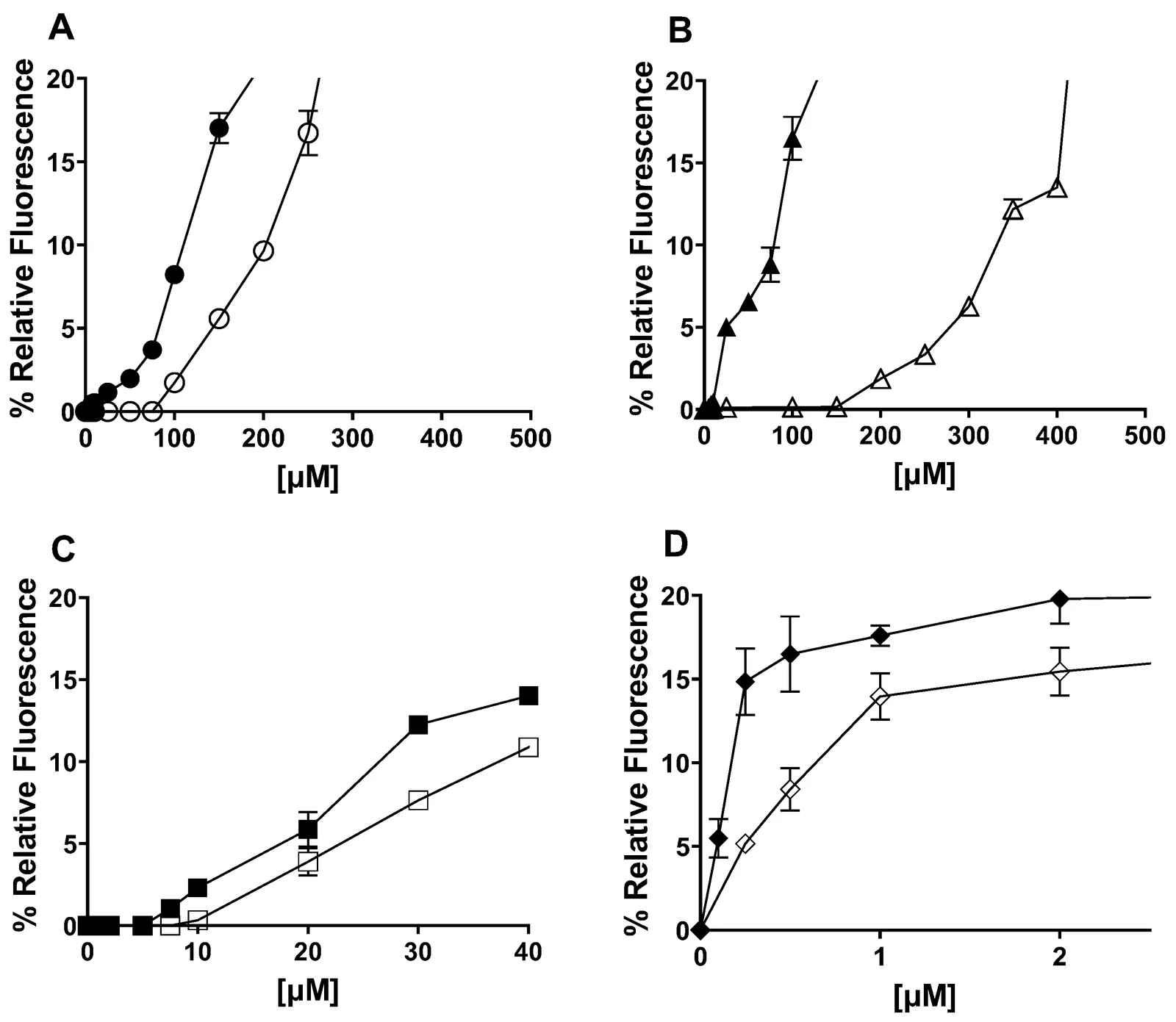
Relative fluorescence at 430 nm (excitation 358 nm) according to the concentration of Resv-4′-LA, Resv-4′-DHA, linoleic acid (LA), and docosahexaenoic acid (DHA) in the absence and in the presence of hydroxypropyl-β-cyclodextrins (HP-β-CDs) in 100 mM sodium phosphate buffer (PBS) pH 7.0 and 35 °C. (**A**) 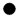 LA, 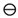 0.25 mM HP-β-CDs/LA; (**B**) 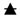 DHA, 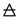 0.25 mM HP-β-CDs/DHA; (**C**) 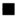 Resv-4′-LA, 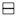 0.25 mM HP-β-CDs/Resv-4′-LA; and (**D**) 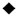 Resv-4′-DHA, 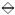 0.25 mM HP-β-CDs/Resv-4′-DHA.

LipoResv (Resv-4′-LA and Resv-4′-DHA) behaved like fatty acids in an aqueous solution, forming micellar aggregates due to their amphiphilic nature (Figure 1). Moreover, in the presence of CDs, inclusion complexes were formed above a concentration of fatty acid (Figure 2A,B) or LipoResv (Figure 2C,D), and an equilibrium was established between micellar aggregates, free and complexed LipoResv or fatty acids [30], as was previously described by López-Nicolás et al. for unsaturated fatty acids [30,31].

The CMC calculation through the measurement of fluorescence by means of a probe such as diphenylhexatriene (DPHT) [31] was the methodology used to study the behaviour of the aggregates at increasing concentrations of LipoResv and the formation of inclusion complexes with CDs, although there are other methodologies to determine the CMC that are also valid [24], such as DLS [32], conductivity, surface tension [26], etc.

### 2.1. LipoResv CMC

In an aqueous solution, the point at which aggregates start to form is specific for each compound and the assay conditions. As can be seen in Figure 2, Resv-4′-LA and Resv-4′-DHA (Figure 2C,D), as well as their respective fatty acids, linoleic acid (LA), and docosahexaenoic (DHA) (Figure 2A,B), increased the relative fluorescence, indicating the formation of aggregates above a certain critical concentration, this being the CMC.

The free fatty acids LA (Figure 2A) and DHA (Figure 2B) needed a higher concentration than their respective LipoResv (Figure 2C,D) to form micelles in the aqueous solution; that is, the CMC_0_ for LA or DHA, were higher than those obtained for Resv-4′-LA and Resv-4′-DHA. The CMC_0_ at pH 7.0 for LA and Resv-4′-LA were 43 and 6 µM, respectively, and in the case of DHA and Resv-4′-DHA they were 70 and 0.001 µM, respectively. These results, in which the CMC_0_ decreased 7 and 70,000-fold for Resv-4′-LA and Resv-4′-DHA, respectively, should be explained by the substitution of the polar head of the fatty acid with the resveratrol molecule, resulting in a new significantly more hydrophobic compound.

Comparing Resv-4′-LA and Resv-4′-DHA, the CMC_0_ of Resv-4′-LA was 6000-fold higher than the CMC_0_ of Resv-4′-DHA, indicating a much lower aqueous solubility of Resv-4′-DHA. The CMC_0_ can be considered as a measure of the self-association tendency of each amphiphilic compound and depends on several parameters. In general, the more surface active the amphiphilic compound, the higher the tendency for micellization and, therefore, the lower its CMC_0_. Therefore, the longer the total length of the hydrocarbon chain of a fatty acid, the lower the CMC [28,31,33].

As has been described in the literature [31], the incorporation of CDs into a fatty acid solution yield to an increase in the CMC value, due to the complexation of fatty acid in the internal cavity of the CDs. This CMC change depends on the capability of each specific type of CD to complex each specific fatty acid. To study the effect of the addition of CDs to aqueous solutions of LipoResv, different types of β-CDs were analyzed (β-CDs, HP-β-CDs, and methyl-β-CDs) by computational simulations. The results obtained showed that HP-β-CDs were the most optimal for the LipoResv encapsulation (Table S1).

CDs are cyclic oligosaccharides composed of glucose units linked via α-1,4-glycosidic bonds. Naturally occurring CDs (α-, β-, and γ-CDs) consist of six, seven, and eight glucose units, respectively, resulting in hydrophobic cavities of varying dimensions. The size of the cavity directly influences their ability to form inclusion complexes with guest molecules. To enhance their aqueous solubility and complexation properties, CDs have been chemically modified, commonly through the introduction of hydroxyl or methyl groups.

These structural features are critical for modulating their interaction with amphiphilic molecules, as demonstrated in our findings and consistent with the previous literature [34]. Such interactions can significantly influence micellization behaviour and consequently alter the CMC of aggregation-prone compounds. Our findings are consistent with the thermodynamic data reported by De Lisi et al., who provided direct calorimetric evidence of interactions between both native and modified CDs and micellar systems. Their study demonstrated that β-CDs and HP-β-CDs form 1:1 and 1:2 host–guest complexes with perfluorinated surfactants, and that these interactions significantly influence micellization by modifying the CMC through complexation equilibria and micelle–CDs interactions [35].

The incorporation of HP-β-CDs in the reaction medium yielded an increase of CMC_0_ in all cases, indicating an increase in the aqueous solubility for both free fatty acids (Figure 2A,B) and LipoResv (Figure 2C,D), with the increase being more pronounced in the case of free fatty acids (Figure 2A,B). The addition of 0.25 mM of HP-β-CDs increased the CMC of LA from 43 to 136 µM (3.2-fold) and in the case of DHA from 70 to 185 µM (2.6-fold). However, in the case of LipoResv, the increase in CMC was lower, from 6 to 8.8 µM (1.5-fold) in the case of Resv-4′-LA and from 0.001 to 0.002 µM (2-fold) in the case of Resv-4′-DHA.

These increases in CMC_0_ values were due to the complexation process of fatty acids and their respective LipoResv in the hydrophobic cavity of HP-β-CDs, as described previously by Matencio et al. for conjugated LA [36]. Importantly, the type and degree of CDs substitution not only affect the extent of encapsulation and micellization, but also the structural features of the guest molecule, which are also critical. In the case of LipoResv, variations in the alkyl chain length and degree of unsaturation alter the compound’s hydrophobicity, which in turn modulates its affinity for the CDs’ cavity and its self-assembly behaviour. The complexation process is directly related to the solubility of the compound. An increase in the hydrophobicity of a compound leads to an increase in its affinity to complex in the hydrophobic cavity of CDs, but it simultaneously implies an increase in its tendency to form micelles [27,33]. As demonstrated in our results and supported by thermodynamic studies from Ondo D. (2020), both the nature of the CDs and the physicochemical properties of the guest molecule significantly influence the equilibrium between inclusion complex formation and micellization, highlighting the competing roles of solubilization and aggregation in these systems [37].

**Figure 3:**
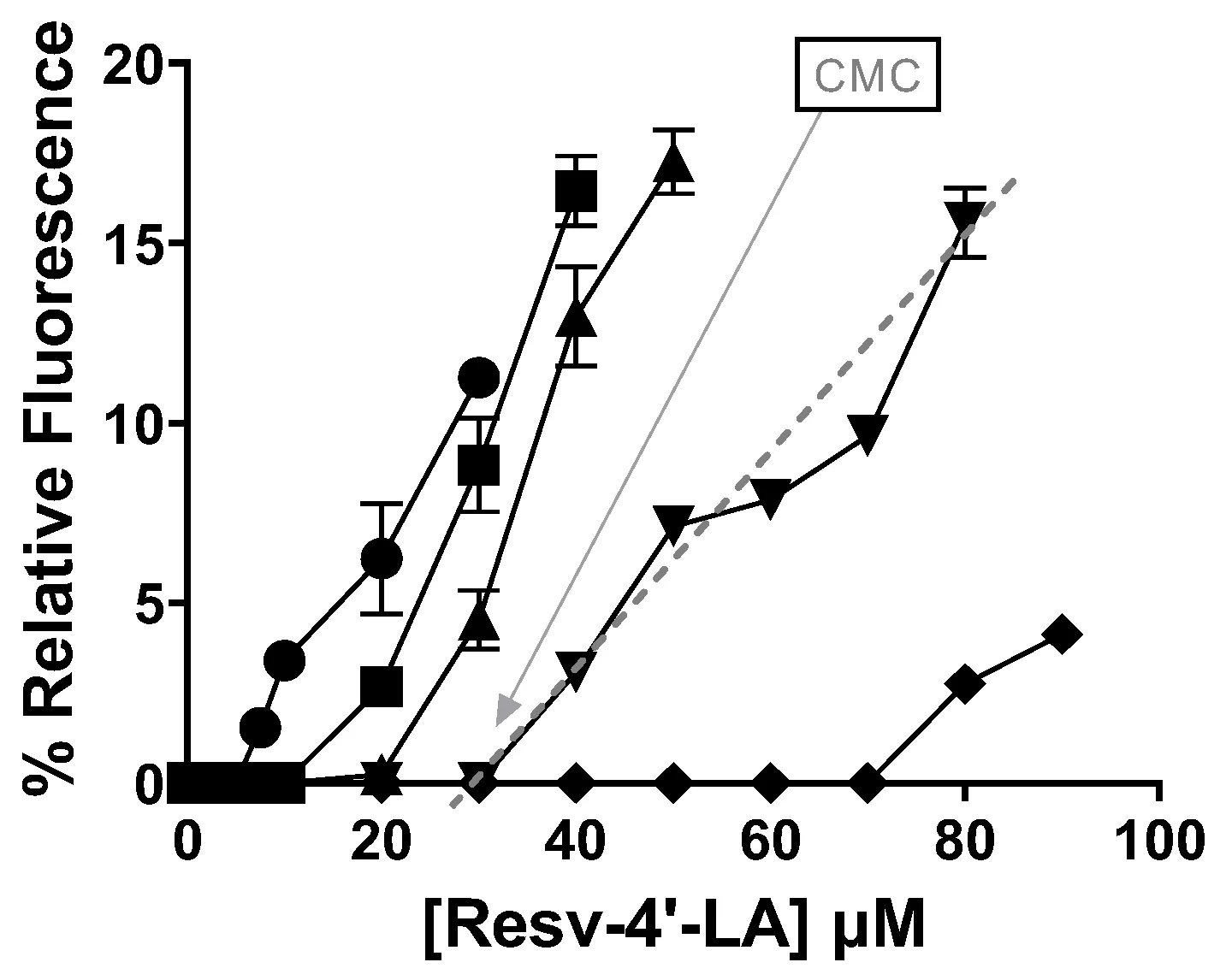
Influence of HP-β-CDs concentration in the relative fluorescence for Resv-4′-LA at pH 7.0 and 35 °C: 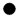 0 mM HP-β-CDs; 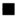 1 mM HP-β-CDs; 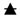 2 mM HP-β-CDs; 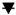 5 mM HP-β-CDs; and 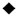 10 mM HP-β-CDs.

Figure 3 shows a detailed representation in which the relative fluorescence increased with the HP-β-CDs concentration in the reaction medium, clearly indicating a delay in the micelle formation by Resv-4′-LA and, thus, in the CMC due to the complexation of Resv-4′-LA in the hydrophobic cavity of CDs. These results align with those reported by other research groups on fatty acids, confirming their dual behaviour in the presence of CDs as a function of concentration [31].

Although the CMC is often reported as a single numerical value, it is not a sharply defined quantity. In practice, micellization occurs over a concentration range, and the value obtained can vary depending on the experimental method employed [24], leading to challenges in its accurate determination [29]. Furthermore, as previously observed, the molecular structure of the amphiphilic compounds is not the sole determinant of the CMC. Owing to the entropically driven nature of micelle formation, the CMC is also influenced by several environmental factors [7,23] including ionic strength, pH, and temperature, as will be further discussed below.

### 2.2. Stoichiometry and Kc of the Complexes LipoResv and CDs

**Figure 4:**
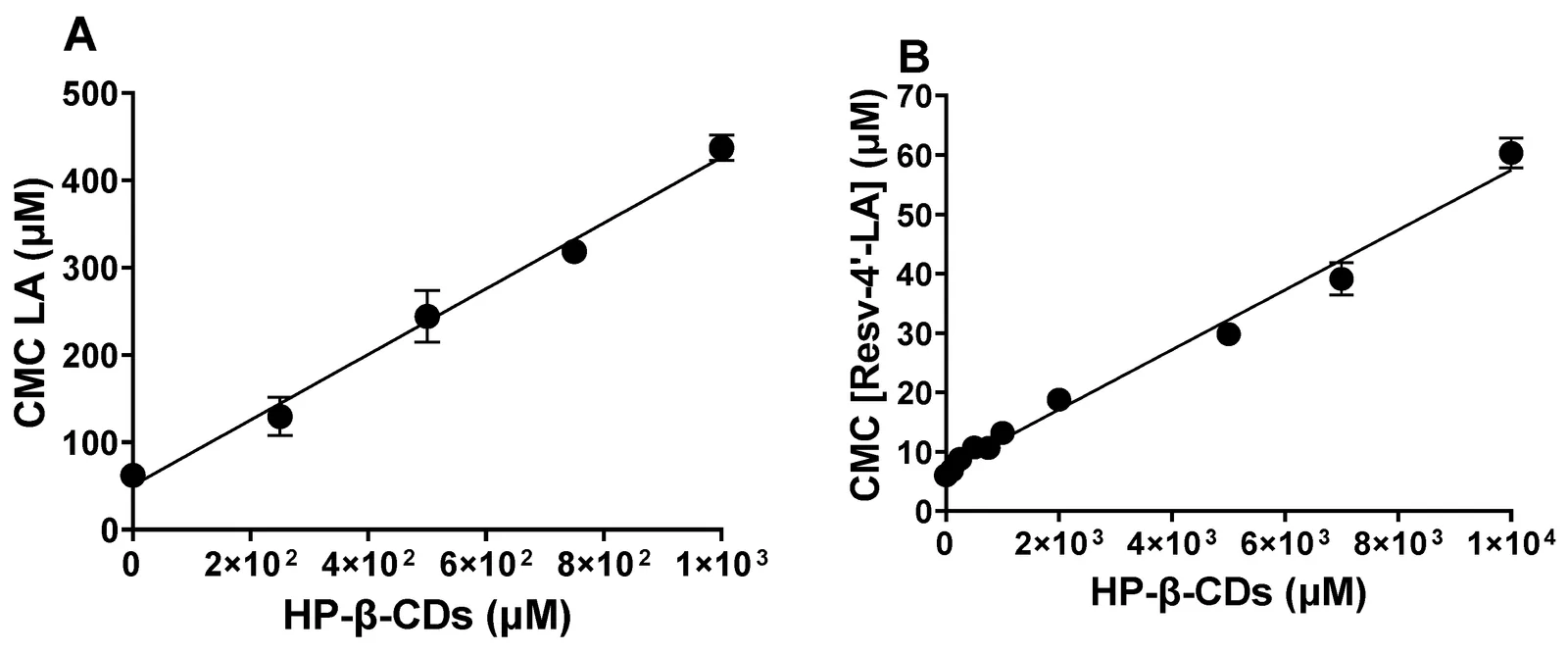

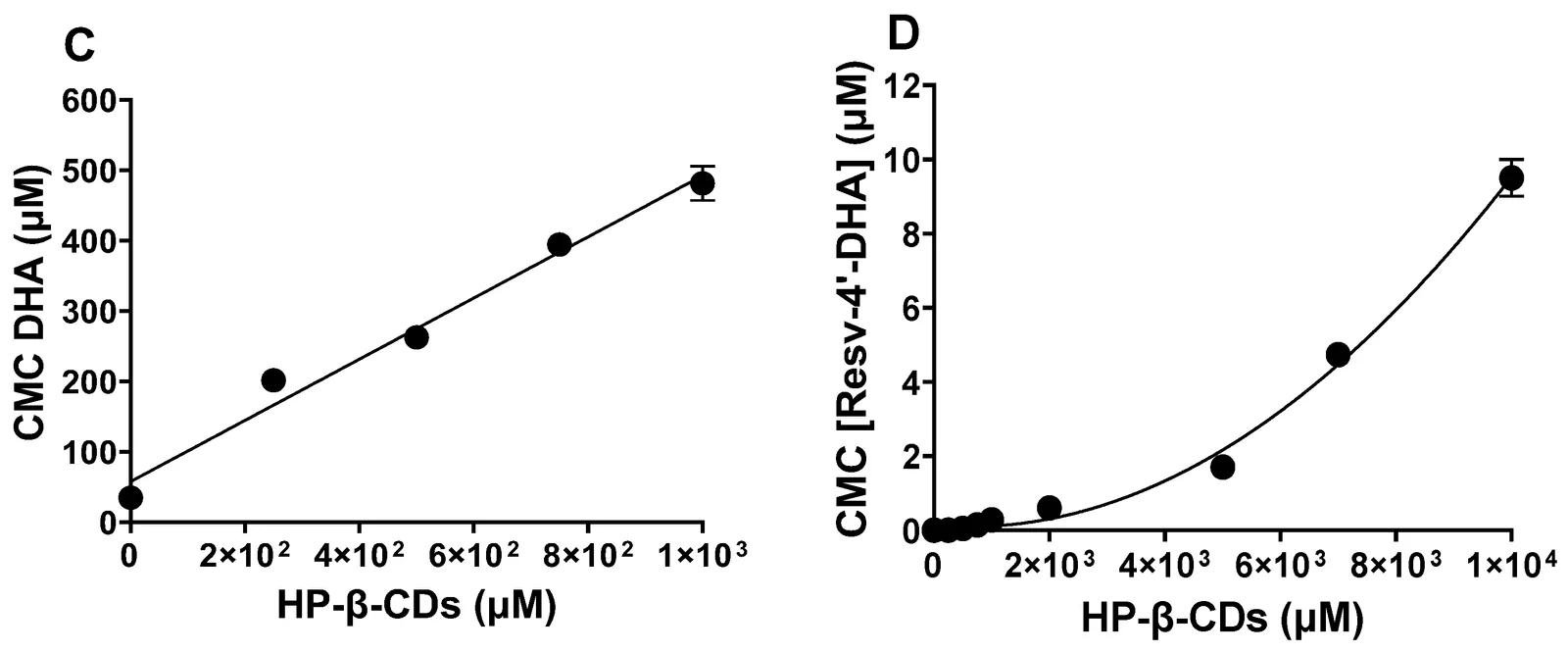
Effect of HP-β-CDs concentration in the critical micellar concentration (CMC) value of LipoResv and its fatty acids at 35 °C and pH 7.0. (**A**) LA, (**B**) Resv-4′-LA, (**C**) DHA, and (**D**) Resv-4′-DHA.

By studying the CMC behaviour of Resv-4′-LA and Resv-4′-DHA with increasing concentrations of HP-β-CDs, the stoichiometry and the Kc value for the complexation processes were determined. As can be seen in Figure 4, the CMC increased with HP-β-CDs concentration in all cases, LA (Figure 4A), Resv-4′-LA (Figure 4B), DHA (Figure 4C) and Resv-4′-DHA (Figure 4D), indicating that the complexation of fatty acids and LipoResv in the hydrophobic cavity of CDs delayed micelles formation.

In the case of Resv-4′-LA, a linear dependence of the CMC with HP-β-CDs concentration was observed (Figure 4B), as in the case of LA (Figure 4A), indicating a 1:1 stoichiometry for the complexation process in both cases. The complexation constant (Kc) value for Resv-4′-LA was calculated by a linear regression to Equation 1, and the value obtained was 720 M^−1^ at pH 7.0 and 35 °C. Comparing the results obtained for Resv-4′-LA (Figure 4B) and its fatty acid counterpart (LA) (Figure 4A) also revealed a 1:1 stoichiometry consistent with results reported previously by other authors [30]. When the Kc between LA and HP-β-CDs was calculated, a value of 7432 M^−1^ was obtained. This value was 10-fold higher than that obtained between Resv-4′-LA and HP-β-CDs, indicating that the complexation between LA and HP-β-CDs is much more stable than between Resv-4′-LA and HP-β-CDs at the same conditions.

Although the most frequent stoichiometry described in the literature for the complexation with HP-β-CDs is 1:1, some molecules may also form 1:2 complexes [21,38].

**Scheme 1:**
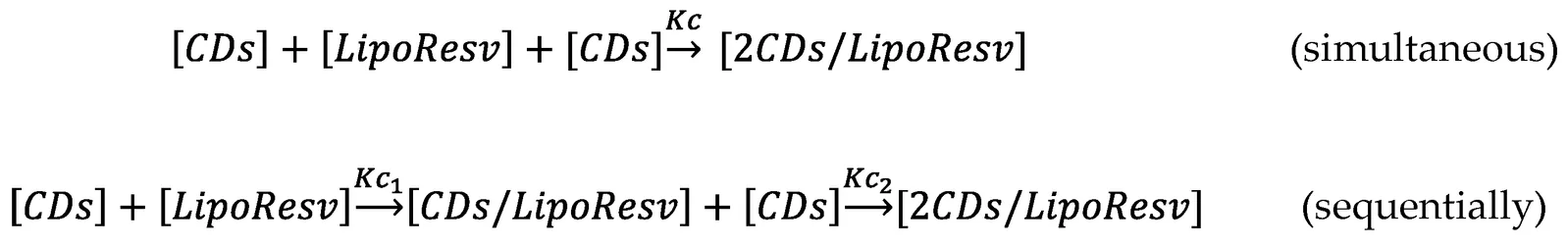
Equations define the complexation process of Resv-4′-DHA with HP-β-CDs, when it’s done in one step (simultaneous) or in two steps (sequential).

When the complexation process of Resv-4′-DHA was analyzed, it could be observed that the CMC also increased with the HP-β-CDs concentration, but clearly in a nonlinear way (Figure 4C), indicating a probably 1:2 stoichiometry for the complexes formed. That is, each molecule of Resv-4′-DHA could be complexed by two molecules of HP-β-CDs. This coupling process could occur simultaneously with a single Kc value or sequentially with two complexation constants (K_1:1_ and K_1:2_) (Scheme 1).

Fitting the data presented in Figure 4D to Equation (4), the results indicated that the coupling process between Resv-4′-DHA and HP-β-CDs occurs sequentially, resulting in two equilibrium constants: K_1_ = 17 M^−1^ and K_2_ = 0.18 M^−1^. The fact that the value of K_2_ was lower than that for K_1_ could be due to the complexation process which was produced by a stepwise binding energy where the coupling of the first HP-β-CDs had a moderate stability, and the second one’s binding energy was reduced due to the most hydrophobic regions of the Resv-4′-DHA already being shielded by the first HP-β-CDs, leaving weaker interactions for the second HP-β-CDs. Although relatively low, it is not uncommon for K_2_ to be smaller than K_1_. The formation of the 1:2 complexes proceed through an initial 1:1 complex formation, which can restrict the spatial orientation and available surface area for the binding of the second CDs molecule.

Triamchaisi et al., in 2023 [39], observed that both 1:1 and 2:1 stoichiometries of β-CDs with cannabidiol and tetrahydrocannabinol were feasible. The 2:1 complex exhibited more favourable complexation energies and greater chemical stability compared to the 1:1 complex. However, the Kc for the 2:1 complex was lower than that observed for the 1:1 complex [39].

In the case of DHA, a linear dependence between CMC and HP-β-CDs was observed, indicating a 1:1 stoichiometry between DHA and HP-β-CDs (Figure 4C), in contrast to the nonlinear dependence observed for Resv-4′-DHA. Fitting the experimental data with a linear regression, the Kc value obtained was 7557 M^−1^. This value was like that obtained for LA and HP-β-CDs, indicating that this type of modified CDs formed complexes with a similar stability in both cases, LA and DHA, probably due to their similar aqueous solubility (CMC of LA 50.5 μM and CMC of DHA 57.5 μM).

While it is evident that an increasing CDs concentration elevated the CMC, determining Kc proves challenging due to different factors: The significant variability in CMC values arising from the equilibrium between CDs and LipoResv describing CDs complexes with 1:1 and 1:2 stoichiometry [40]; the presence of various conformations [41,42]; the formation of aggregates with different sizes as a result of CDs incorporation [43]; and the difficulty in establishing a stable S_0_ in highly insoluble compounds [14].

Although the literature commonly reports that CDs exhibit a greater affinity toward more hydrophobic compounds [10], this trend has not been observed in the present study. This discrepancy highlights the importance of evaluating each compound individually, as CDs–guest interactions could be influenced by factors beyond hydrophobicity, such as molecular size, geometry, and the distribution of functional groups of guest molecules. Furthermore, it should be considered that the determination of the CMC is inherently complex, particularly in systems containing CDs. As demonstrated by Yun-Bao Jiang and Xiu-Juan Wang, the formation of CDs inclusion complexes can significantly alter the aggregation behaviour of surfactants. Specifically, the 1:1 β-CDs/surfactant complex may act as an alternative hydrophobic core, promoting surfactant monomer aggregation even at concentrations below the conventional CMC [44].

### 2.3. Particle Size in Relation to CMC and Complex Formation

**Figure 5:**
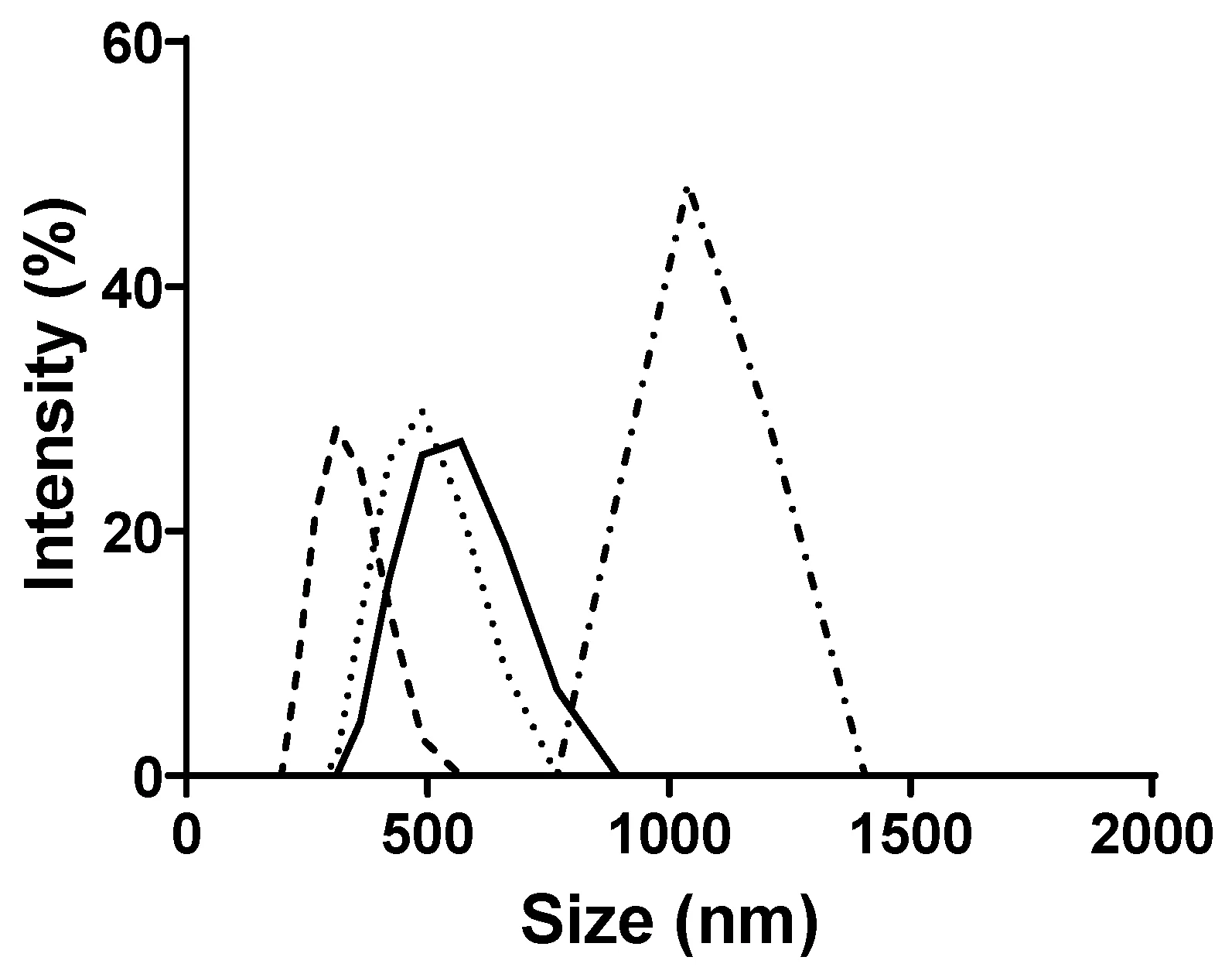
Particle size (nm) by dynamic light scattering (DLS): Resv-4′-LA 10 µM (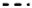), Resv-4′-LA 50 µM (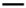), Resv-4′-DHA 10 µM (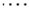), and Resv-4′-DHA 50 µM (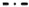) in PBS at 35 °C and pH 7.0.

DLS was employed to estimate the hydrodynamic diameter of the micelles and to assess the polydispersity index (PDI) of the system at 35 °C and pH 7.0 for both Resv-4′-LA and Resv-4′-DHA (Figure 5).

The average micelle size and the PDI were found to vary as a function of LipoResv and HP-β-CDs concentration, indicating a heterogeneous population of particle size.

The results presented in Figure 5 demonstrated that the particle size increased with the LipoResv concentration. At a fixed concentration of 10 µM of LipoResv, the particle size for Resv-4′-LA and Resv-4′-DHA were 438 (Figure 5, 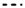) and 600 nm (Figure 5, 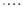), respectively. However, when the LipoResv concentration increased to 50 µM, the particle size for Resv-4′-LA increased to 622 nm (Figure 5, 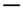), (1.4-fold), and for Resv-4′-DHA, it increased to 2989 nm (Figure 5, 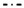), (5-fold). These findings suggested that Resv-4′-LA exhibited a greater aqueous solubility compared to Resv-4′-DHA. This interpretation aligns with the CMC_0_ values obtained in the absence of CDs, which were 6 µM for Resv-4′-LA (Figure 2C) and 0.001 µM for Resv-4′-DHA (Figure 2D). The lower CMC_0_ of Resv-4′-DHA (6000-fold) indicated that micelle formation occurred at a much lower concentration than in the case of Resv-4′-LA due to the lower aqueous solubility of Resv-4′-DHA (Table 1).

The presence of HP-β-CDs may lead to their inclusion within the micellar system, thereby increasing the overall size [43,45]. It is also possible that CDs complexes self-assemble into larger aggregates, as previously reported in the literature [44]. Although an increase in particle size was observed in our experimental system, the incorporation of HP-β-CDs may contribute to a reduction in micellar aggregation by encapsulating LipoResv [45].

To evaluate the colloidal stability of the particles analyzed by DLS, 50 µM Resv-4′-LA solutions were prepared in the presence and absence of HP-β-CDs, initially characterized and then stored at room temperature in the dark for 24 h before a second DLS measurement was performed. Subsequent particle size measurements revealed an increase. Initially, the particle size of Resv-4′-LA in the absence of HP-β-CDs was 622 nm, increasing to 933 nm after 24 h (1.5-fold). Moreover, in the presence of HP-β-CDs, the initial particle size was 894 nm, which increased to 1073 nm (1.2-fold) after 24 h of storage.

### 2.4. Influence of pH, Temperature, and Ionic Strength on LipoResv CMC and Complexation Process

As the micellization tendency of amphiphilic molecules and complexation in CDs processes could be affected by temperature, pH, and ionic strength, the effect of these parameters for Resv-4′-LA and Resv-4′-DHA in the presence of HP-β-CDs were studied. Although changes in temperature (15, 25, and 35 °C), pH (2.0 and 7.0), and salt concentration (sodium phosphate buffer (PBS) 100 mM and MilliQ water) did not cause changes in the CMC’s profile, the concentration of HP-β-CDs, linear in the case of Res-4′-LA and nonlinear in the case of Res-4′-DHA, produced variations in CMC and Kc values for both Res-4′-LA and Res-4′-DHA (Table 2).

As can be seen in Table 2, the acidification of the pH of the reaction medium (pH 7.0 to pH 2.0) involved a decrease in CMC_0_ in both cases. For Resv-4′-LA, the CMC_0_ decreased from 6 µM at pH 7.0 to 0.54 µM at pH 2.0, and from 0.001 to 5 × 10^−4^ µM in the case of Resv-4′-DHA, indicating a lower solubility of LipoResv at an acidic pH in both cases, but with a more pronounced decrease in the case of Resv-4′-LA. Moreover, a decrease in the temperature also affected to the CMC_0_ values, diminishing from 6 µM at 35 °C to 0.27 µM at 25 °C and 0.14 µM at 15 °C, in the case of Resv-4′-LA; and from 0.001 to 6 × 10^−4^ and 3 × 10^−4^ µM, respectively, in the case of Resv-4′-DHA. The ionic strength of the reaction medium interfered with micelle formation, decreasing the CMC_0_ of Resv-4′-LA from 6 to 0.11 µM and, in the case of Resv-4′-DHA, from 0.001 to 5 × 10^−4^ µM when the reaction medium was MilliQ water.

The decrease observed in CMC_0_ with pH, temperature, and ionic strength also implied changes in the Kc values for both LipoResv in the presence of HP-β-CDs. For Resv-4′-LA, a decrease in the pH lead to an increase in the Kc value from 720 to 4264 M^−1^. In the case of Resv-4′-DHA, where 1:2 complexes were formed, the K_1_ value increased from 17 to 97 M^−1^ and K_2_ from 0.18 to 0.45 M^−1^ (Table 2). When the temperature decreased from 35 to 25 and 15 °C, the Kc values increased from 720 to 8157 and 10,432 M^−1^, respectively, for Resv-4′-LA; from 17 and 0.18 M^−1^ for K_1_ and K_2_, respectively, to 787 and 0.1 M^−1^ at 25 °C; and to 898 and 0.33 M^−1^ at 15 °C in the case of Resv-4′-DHA. In the absence of salt in the reaction medium, Kc values increased for LipoResv. In the case of Resv-4′-LA, the Kc value increased from 719 to 42,535 M^−1^ and from 17 to 707 M^−1^ in the K_1_ value and from 0.18 to 0.33 M^−1^ in the K_2_, in the case of Resv-4′-DHA (Table 2).

These results obtained with the pH changes could be explained by the stabilization of the hydrocarbon chain of the fatty acid in an acidic media. By lowering the pH, the carboxyl group of the fatty acid and the -OH group of the HP-β-CDs remain intact and favour the formation of hydrogen bonds between LipoResv and the hydrophobic cavity of the HP-β-CDs. Moreover, the non-deprotonated form of the LipoResv is more hydrophobic, thus favouring its entry into the internal cavity of the HP-β-CDs through hydrophobic interactions. When a molecule is in its neutral form, it exhibits an increased hydrophobic character with respect to its ionic form, which reduces electrostatic interactions with CDs, providing a better fit within their hydrophobic cavity [46,47]. Furthermore, an increase in temperature leads to a rise in molar conductivity, which is attributed to a reduction in viscosity and an enhancement in ion mobility within the solvent, leading to an increase in the CMC_0_ value with temperature [46]. This behaviour varies depending on the specific molecule under research. At a high temperature, the Kc values generally decrease [47]; however, Kinart, in 2023, observed that in the case of HP-β-CDs and dodecanoic acid, the Kc values increased initially with the temperature, reaching maximum value at 25 °C and decreasing after that [48].

**Figure 6:**
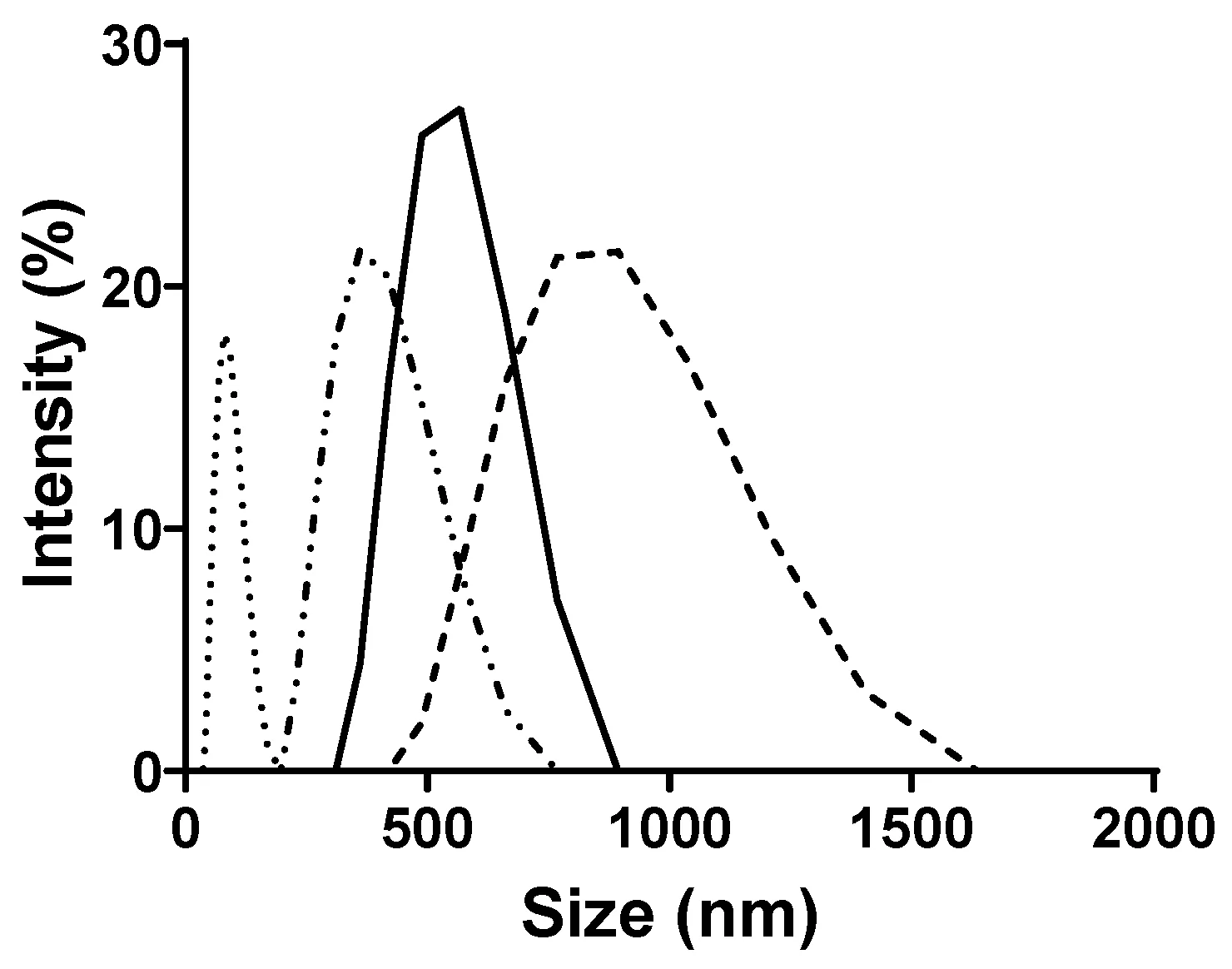
Particle size (nm) of Resv-4′-LA 50 µM (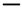), Resv-4′-LA 50 µM with 10 mM HP-β-CDs (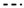) in PBS, Resv-4′-LA 50 µM (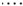), and Resv-4′-LA 50 µM with 10 mM HP-β-CDs (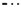) in MilliQ water at 35 °C and pH 7.0.

Hinze also described that the presence of salt in the reaction medium neutralizes the charge of micelles, thereby promoting their stability and formation. However, these changes may influence the system in different ways, depending on the specific compound involved and the characteristics of the surrounding medium [46]. Ionic strength plays a critical role not only in the initiation of micelle formation but also in the complexation behaviour with HP-β-CDs. The presence of salts markedly affects particle size. Particle size decreased substantially in the PBS medium relative to MilliQ water. Specifically, for 50 µM Resv-4′-LA, the particle size observed in the PBS medium was 600 nm, whereas in MilliQ water, it was significantly reduced to 82 nm. Similarly, upon the addition of 10 mM HP-β-CDs, the particle size decreased from 894 nm in PBS to 383 nm in MilliQ water (Figure 6). Moreover, as shown in Table 2, the CMC_0_ is higher in 100 mM PBS than in MilliQ water, indicating that the micelle formation began at lower LipoResv concentrations in MilliQ water.

The variation observed in the Kc as a function of different physio-chemical parameter values underscores the high sensitivity of host–guest interactions within reaction media, as highlighted previously by Hinze [46]. This intrinsic complexity offers the opportunity to finely modulate the encapsulation and release dynamics of active compounds such as LipoResv. Such tunability is particularly relevant for applications in controlled release systems, functional food formulation, and the design of molecular sensors.

Literature reports indicate that ionic strength can significantly influence both the micellization process and the resulting particle size. In the PBS medium, a higher concentration of LipoResv was required to initiate micellization. However, once micelles were formed, the presence of ions in the medium promoted micellar aggregation, leading to an increase in particle size. In contrast, the absence of ions in MilliQ water favours the formation of smaller micelles [49].

### 2.5. Computational Models

**Figure 7:**
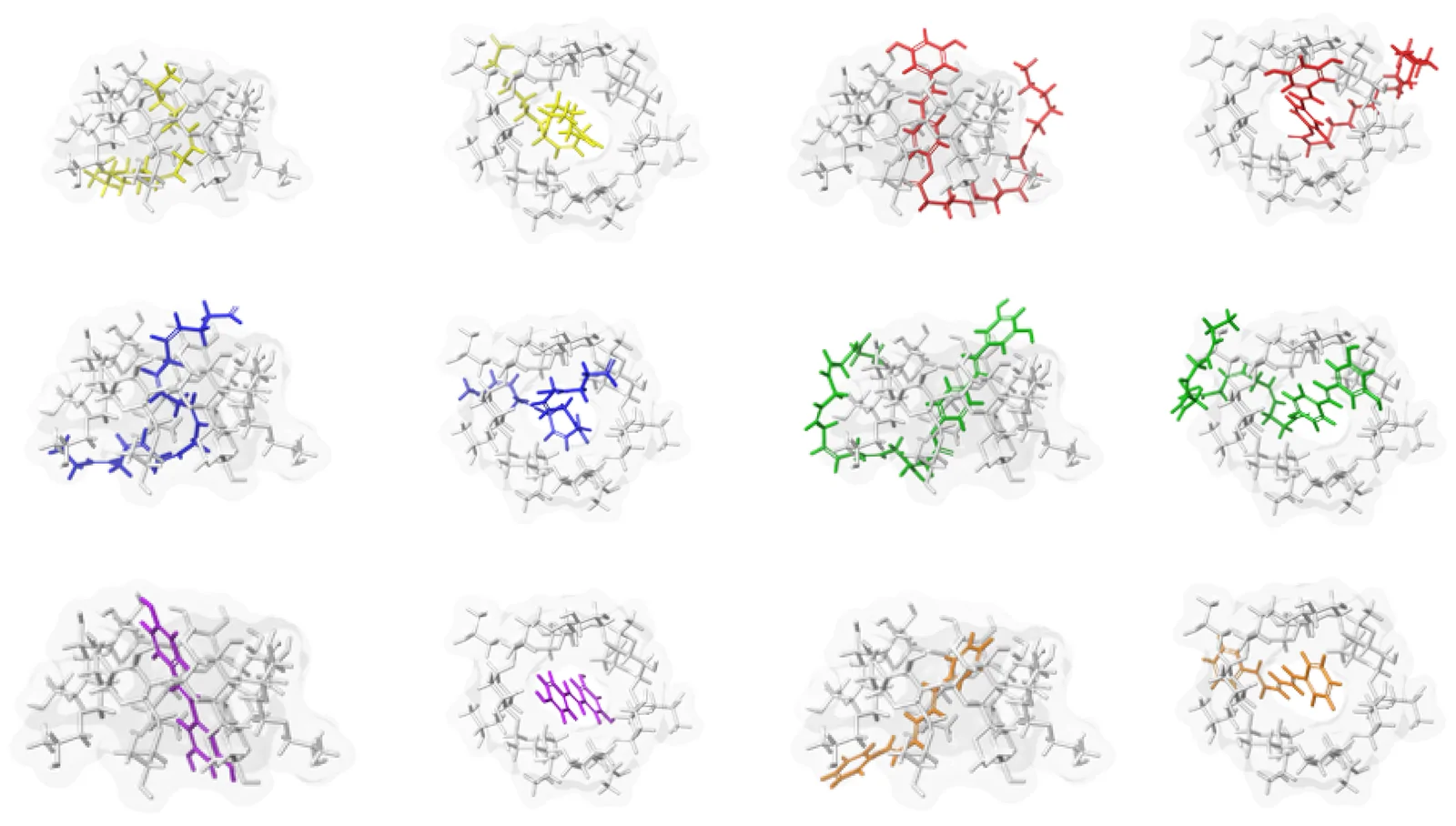
Docking poses corresponding to the most favourable host–guest interaction energies for HP-β-CDs inclusion complexes with six guest molecules: resveratrol (purple), diphenylhexatriene (DPHT) (orange), LA (yellow), DHA (blue), Resv-4′-LA (red), and Resv-4′-DHA (green). Cyclodextrins (CDs) are shown as stick-and-surface representations in grey, whereas guest molecules are rendered in coloured-stick format. Two perspectives are shown for each complex: a front view and a lateral view, highlighting both internal accommodation and external extension of the guest structures.

The performed simulations hint that HP-β-CDs form stable inclusion complexes with a range of structurally diverse guests, including natural fatty acids (i.e., LA and DHA), their LipoResv (i.e., Resv-4′-LA and Resv-4′-DHA), and small model compounds such as resveratrol and DPHT. As illustrated in Figure 7, docking simulations show the consistent insertion of the hydrophobic portions of all guests into the CDs’ cavity. Even though the early docking results suggested compatibility across all systems, a closer inspection of the inclusion poses reveals dissimilarities depending on the size and nature of the guest molecule.

Smaller compounds, such as resveratrol and DPHT, are fully accommodated within the internal cavity of HP-β-CDs. In contrast, the unmodified fatty acids LA and DHA exhibit the partial insertion of their hydrophobic tails into the CDs’ cavity. Their relatively flexible carbon chains are located along the apolar axis of the host, optimizing van der Waals contacts with the interior walls while leaving the carboxylic acid headgroup closer to the cavity entrance. These poses remain energetically favourable and are consistent with typical inclusion patterns reported for medium-chain fatty acids [50]. The most pronounced differences are observed for the LipoResv, Resv-4′-LA, and Resv-4′-DHA. Due to their larger molecular size, these molecules exceed the spatial capacity of the CDs’ cavity. In their optimal docking poses, the resveratrol moiety is anchored within the CDs’ core, while the long hydrocarbon chains are located beyond the cavity opening and extend outward into the solvent. This arrangement reflects a compromise between maximizing the favourable host–guest interactions and minimizing steric clashes and is further influenced by geometric constraints arising from unsaturation in the fatty acid chains, especially in the DHA, where kinks introduced by multiple double bonds reduce packing efficiency within the inner cavity.

Figure 6 captures these variations in encapsulation geometry, highlighting how the interplay between molecular size, flexibility, and hydrophobic–hydrophilic balance governs the inclusion mode. This qualitative analysis was further supported by binding free energy calculations. According to the Molecular Mechanics/Generalized Born Surface Area (MM-GBSA) approach, HP-β-CDs exhibited a clear preference for larger and functionalized guests. The largest interaction energy (the most negative) was obtained for Resv-4′-LA (−64.36 kcal/mol), followed by Resv-4′-DHA (−58.34 kcal/mol), DHA (−51.05 kcal/mol), LA (−47.24 kcal/mol), DPHT (−47.38 kcal/mol), and, finally, resveratrol (−45.52 kcal/mol). These results suggest that LipoResv significantly enhances the binding affinity, likely due to an increased hydrophobic surface area and improved van der Waals complementarity within the HP-β-CDs’ cavity, in line with previous studies on amphiphilic guest molecules [51]. The numerical ranking also implies that both the native fatty acids and their LipoResv exhibited stronger binding than the fluorophore DPHT. These results confirm the departure of DPHT from the CDs cavity upon complexation with natural and modified fatty acids. Moreover, the original resveratrol showed a less intense interaction (less negative binding energy), highlighting how conjugation with LA or DHA improves complexation efficiency with HP-β-CDs.

However, our experimental data offer a contrasting picture. The experimentally determined Kc for LA was 7432 M^−1^, while that for Resv-4′-LA was only 719 M^−1^, implying that LA forms a significantly more stable inclusion complex under identical conditions. This discrepancy suggested that absolute MM-GBSA binding energies alone may not provide a fully accurate representation of complex stability in a solution, particularly when comparing ligands of different size and flexibility. To address this, we evaluated the ligand efficiency (LE), defined as the binding energy per heavy atom of the guest molecule. This normalized metric allows for a fairer comparison of guests with varying molecular size, which is our case [52,53].

Interestingly, LE values altered that ranking. LA showed the highest ligand efficiency (−2.362 kcal/mol per heavy atom), followed by DHA (−2.127 kcal/mol), Resv-4′-LA (−1.788 kcal/mol), and Resv-4′-DHA (−1.459 kcal/mol). These data suggest that although the LipoResv exhibit stronger absolute interactions, their per-atom binding efficiency is lower due to the impact of entropic and packing effects. This trend aligns more closely with the experimental Kc values, where LA outperformed Resv-4′-LA by an order of magnitude. A similar trend is observed between Resv-4′-LA (Kc = 719 M^−1^) and Resv-4′-DHA (Kc = 17 M^−1^) (Table 1), confirming the superior performance of the Resv-4′-LA, both experimentally and computationally (in LE terms). The performed simulations hint at the importance of implementing both absolute and normalized binding metrics in the theoretical assessment of inclusion complexes. While the MM-GBSA highlights the cumulative interaction strength, LE offers a more balanced view for molecular packing efficiency in CDs-related systems. This dual analysis strategy has been employed successfully in the design of host–guest systems and fragment-based drug discovery [52,54] and is particularly valuable here in explaining why smaller, more compact guests such as LA can outperform larger conjugates in real solution conditions (Table S2).

**Figure 8:**
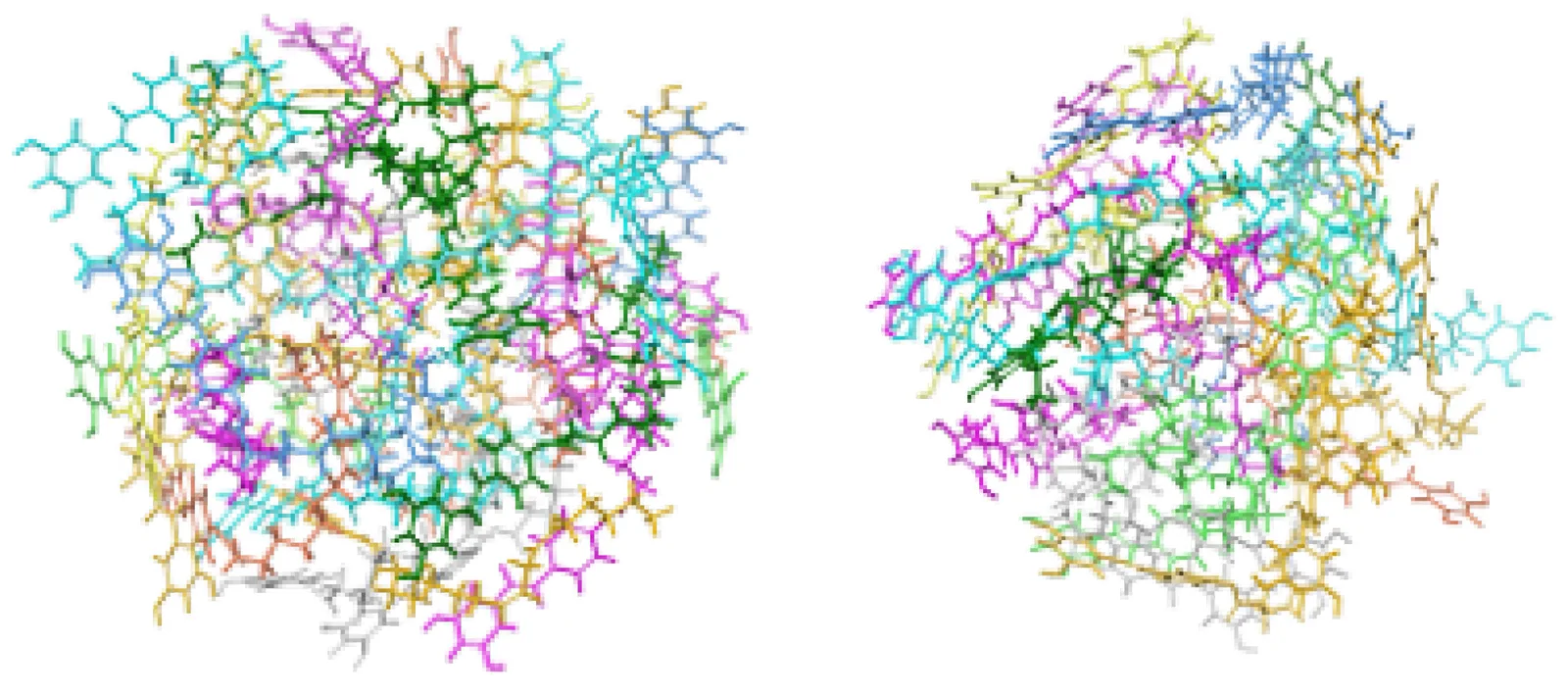
Designed model systems of micellar aggregates for Resv-4′-LA ((**left**), contains 30 monomeric units) and Resv-4′-DHA ((**right**), 24 units). The structures correspond to the clusters extracted from the final 100 ns simulation trajectories associated with the most favourable intermolecular interaction energies, as determined by Molecular Mechanics/Generalized Born Surface Area (MM-GBSA) analysis. Micellar models are shown as coloured sticks.

The computational protocol was finally completed by conducting MD simulations with a focus on assessing the micellization processes. As discussed above, the CMC value might be used as an indirect measure of surface activity and aqueous solubility. Under a defined pH and temperature, this intrinsic property is denoted as CMC_0_ and typically decreases with increasing hydrophobicity or chain length of the molecule [55]. Experimentally, Resv-4′-LA and Resv-4′-DHA, as well as their precursor fatty acids LA and DHA, showed increased relative fluorescence at concentrations above their respective CMC, confirming the formation of colloidal aggregates. As noted, experimental outcomes showed a striking difference between the two LipoResv: the CMC_0_ of Resv-4′-LA was approximately 6000 times higher than that of Resv-4′-DHA, indicating a much lower tendency of Resv-4′-LA to self-associate in an aqueous medium. This discrepancy aligns with their physicochemical profiles—Resv-4′-DHA, bearing a longer and more unsaturated hydrophobic chain, exhibits greater surface activity and, consequently, a lower CMC_0_, as expected from classical amphiphilic behaviour [56]. To complement these findings, atomistic MD simulations were carried out to model the formation and stabilization of micellar aggregates, as illustrated in Figure 8.

The simulations confirmed the spontaneous micellar formations that allow for clustering to extract representative structures. The MM-GBSA interaction energy of a single unit within the micelle was computed next, providing a theoretical estimate of the cohesive strength within the aggregate. It is worth noting that while the MMGBSA method, combined with molecular dynamics (MD), offers a balanced compromise between computational cost and accuracy. Of course, that approach might be associated with errors in estimating the absolute binding free energies, particularly due to its implicit treatment of entropic effects and the finite sampling upon a conformational search. However, the agreement between theoretical and experimental complexation constants validates the relative trends captured by our simulations. Furthermore, the present computational framework provides a valuable mechanistic insight and correctly mimics the inclusion equilibria and micelle-forming capacity of LipoResv compounds.

## 3. Materials and Methods

### 3.1. Materials

HP-β-CDs were provided by AraChem (Tilburg, the Netherlands); according to the manufacturer, the average degree of substitution was 0.6, and the average molecular weight was approximately 1541.46 g/mol. LipoResv (Resv-4′-LA and Resv-4′-DHA) were obtained from the Institute of Biomolecules Max Mousseron (IBMM), Pôle Chimie Balard Recherche, Montpellier (France). Free LA, DHA, DPHT, and tetrahydrofurane (THF) were purchased from Sigma Aldrich (Madrid, Spain). The rest of the chemical compounds used were of analytical grade.

### 3.2. Chemo–Enzymatic Synthesis of LipoResv

Resv-4′-La and Resv-4′-DHA were synthesized according to the previous procedure [5,6] using enzymatic and chemical synthesis starting from resveratrol. The procedure was adapted for gram scale synthesis. In the first step, the supported lipase *Candida antartica* (CALB, Novozyme 435) was provided by Novozymes A/S, (Bagsvaerd, Denmark), and used to introduce the acetyl group regioselectivity at the resveratrol C4′-OH position. The reaction was performed in good yield (85%) without any acetyl derivatives in 3 or 5 positions. Hydroxyls at 3 and 5 positions were then protected by TIPS groups using TIPS-OTf and DIPEA as a base to obtain a 3,5-protected derivative. The acetyl group at the 4′position was deprotected with MeONa in anhydrous methanol and resulted resveratrol-3,5-diTIPS in excellent yield (90–95%). The coupling reactions between the silylated polyphenol and the selected fatty acid (LA or DHA) was initiated using DCC/DMAP as a coupling reagent (74% for LA and 79% for DHA). Final TIPS deprotection by Et_3_N-3HF in dry THF yielded Resv-4′-LA (88%) or Resv-4′-DHA (90%). ^1^H-NMR analysis of the synthesized lipophenols were in accordance with the literature.

### 3.3. Fluorimetric Determination of CMC

The CMC of fatty acids and LipoResv were determined by means of the fluorescence spectroscopy method by Chattopadhyay et al. in 1984 [56]. Increasing concentrations of different types of CDs were added to saturate buffered solutions of fatty acids or LipoResv at different pHs, using DPHT (in THF) as a fluorescent probe. The reaction medium contained 2 mL of 100 mM PBS pH 7.0, MilliQ water (18 MΩ) pH 7.0, and 100 mM sodium borate buffer pH 2.0; 0.89 µM DPHT (supplied in 20 µL of 10% THF solution), 20 µL of fatty acid or LipoResv ethanolic solution, and a given concentration of HP-β-CDs dissolved in the respective buffer or MilliQ water.

The solutions were vortexed and given a N_2_ flush prior to incubation in the dark for 60 min at different temperatures (35, 25, and 15 °C). Fluorescence intensity was measured at 430 nm (excitation wavelength 358 nm) using a SpectraMax iD3 plate reader from Molecular Devices (San Jose, CA, USA), equipped with a thermostat set at an appropriate temperature, using a blank containing all reagents except LipoResv. A detailed summary of the experimental conditions used for solubilization and complexation studies is provided in the Supplementary Information as Table S3.

The CMC was determined graphically by plotting the relative fluorescence percentage vs. fatty acid or LipoResv concentration as the intersection between the post-micellar line fluorescence trend and the pre-micellar baseline. The detailed preparation procedure is described in Supplementary Protocol S1. To assess the precision of the fluorescence measurements, intra- and inter-day variability was evaluated. The corresponding data, including fluorescence intensity values for controls and selected samples at different concentrations, are shown in the Supplementary Information as Table S4.

### 3.4. Determination of Complexation Stoichiometry and Equilibrium Constant Between CDs and Fatty Acid or Liporesv

To characterize the formation of HP-β-CDs/LipoResv or HP-β-CDs/fatty acid inclusion complexes, the apparent CMC was plotted as a function of HP-β-CDs concentration, resulting in a micellar diagram indicating the inclusion complexes’ stoichiometry.

In the case of a post-micellar linear trend, a 1:1 stoichiometry is considered for inclusion complexes formations [25], and the complexation constant (Kc) was calculated by the following equation:

(1) $$CMC=CMC_{0}+Kc\times CMC_{0}[CDs]$$

where CMC_0_ is the critical micellar concentration in the absence of CDs, which is characteristic for each compound at a given pH, temperature, and ionic strength. A linear representation of CMC vs. [CDs] with a regression coefficient close to 1 indicates a 1:1 stoichiometry.

On the other hand, if the post-micellar trend fits a quadratic model, 1:2 stoichiometry is considered [14,21], where the complex is formed by the sequential binding of 2 molecules of HP-β-CDs to 1 molecule of LipoResv or fatty acid, and the Kc are defined as follows:

(2) $$K_{1}=\frac{\left[\mathrm{LipoResv}-\mathrm{CDs}\right]}{\left[\mathrm{LipoResv}\right][\mathrm{CDs}]}$$

(3) $$K_{2}=\frac{\left[\mathrm{LipoResv}-{\mathrm{CD}}_{2}\right]}{\left[\mathrm{LipoResv}-\mathrm{CDs}\right][\mathrm{CDs}]}$$

As highlighted by Lopez-Nícolas et al. in 1995 [57], the fatty acid (or LipoResv in our case) concentration can be replaced by CMC in the presence of CDs and CMC_0_ in their absence. Further details of the fitting procedure used for Kc determination are provided in the Supplementary Information (Supplementary Note S1). Thus, the equilibrium constants K_1_ and K_2_ could be estimated using the following equation:

(4) $$CMC=CMC_{0}+K_{1}CMC_{0}\left[CDs\right]+K_{1}K_{2}CMC_{0}{\left[CDs\right]}^{2}$$

### 3.5. Determination of Particle Size by DLS

The size distribution and PDI of micelles was determined using a Zetasizer Red Ultra equipped with a 10 mW red laser at a scattering angle of 173° (backscatter) at 25 °C. Samples were prepared like in the case of the fluorescent determination of CMC (see Section 3.3) but without the fluorescent probe. To avoid interference from any particles present, all reagents were filtered through a 0.22 µm nylon filter before sample preparation. After that, samples were prepared and incubated at 35 °C for 60 min to stabilize micelle formation. The samples were run in triplicate, and the resulting values represent the average of three runs, each comprising two measurements. Data analysis was performed using the integrated XZ XPLORER software v4.0.0 to calculate particle size distribution.

To further assess the influence of ionic strength on micelle aggregation, CMC was investigated using two different media: MilliQ water and 100 mM PBS at pH 7.0.

### 3.6. Molecular Modelling

Computational models have been implemented to simulate the host–guest encapsulation processes. The initial three-dimensional structures of resveratrol, the fluorophore DPHT, and the unfunctionalized LA and DHA, as well as their modified counterparts, Resv-4′-LA and Resv-4′-DHA, were generated using the Maestro software (https://www.schrodinger.com/platform/products/maestro/ Accessed 30 June 2025) from Schrödinger, LLC (New York, NY, USA) [58]. The HP-β-CD–host model was built using the same software and protocol. All molecular structures were prepared and optimized at a neutral pH (7.0), consistent with experimental conditions, by using the LigPrep module from Schrödinger. This step included protonation state assignment, stereochemistry preservation, geometry minimization using the Optimized Potentials for Liquid Simulations, version 4 force field (OPLS4), and partial charge calculations [59].

Docking simulations were carried out using the Glide software (https://www.schrodinger.com/platform/products/glide/ Accessed 30 June 2025) (standard precision mode), allowing up to 10 poses per ligand to correctly explore conformational space, from Schrödinger, LLC (New York, NY, USA) [60]. The HP-β-CDs’ cavity was treated as a rigid host, while full ligand flexibility was enabled. Interaction energies obtained from docking were further refined through MM/GBSA calculations using the Prime module [61]. These energies offer a more accurate approximation of binding affinities and are reported in Kcal/mol. All computational studies related to the inclusion of fatty acids within CDs cavities were carried out using the same force field, e.g., OPLS4. This force field has been shown to offer improved accuracy for modelling noncovalent interactions and conformational behaviour in host–guest systems, including carbohydrate-based hosts like cyclodextrins and hydrophobic ligands such as fatty acids [62]. The accumulated CPU cost associated with each model system, including docking and post-processing with MM/GBASA refinement, is ca. 10 h/ligand.

In addition to the inclusion complexes, micellar models of functionalized fatty acids were built. These spherical assemblies mimic the amphiphilic nature of the Resv-4′-LA and Resv-4′-DHA conjugates, placing the polar resveratrol moieties toward the micelle surface and the hydrophobic alkyl chains toward the interior. For Resv-4′-LA, a total of 30 units were required to form a stable spherical configuration. In the case of Resv-4′-DHA, the higher degree of unsaturation reduces the number of compatible chains to 24, as larger numbers resulted in central overlap. Micelles were solvated in orthorhombic water boxes, with a 10 Å buffer distance in all directions. MD simulations were then performed using the GPU-accelerated version of Desmond for 100 ns trajectories [63]. The average computational GPU cost is ca. 12 h running in a NVIDIA Quadro RTX 5000 GPU cards from PC Componentes, Murcia, Spain.

## 4. Conclusions

As highlighted by the results, the LipoResv compounds (Resv-4′-LA and Resv-4′-DHA) exhibit significantly greater hydrophobicity compared to their parent fatty acids. This is an enhanced hydrophobicity compared to their parent fatty acids. This enhanced hydrophobicity is reflected in their micellization behaviour, as evidenced by substantially lower CMC_0_ values. These changes arise from the structural modification involving the conjugation of fatty acids to resveratrol, which alters their physicochemical properties. Moreover, this study demonstrates, for the first time, the potential of LipoResv compounds as self-assembling amphiphilic systems capable of forming stable inclusion complexes with HP-β-CDs. The host–guest interactions between LipoResv and HP-β-CDs were found to be highly dependent on the structural features of the guest molecules as well as the physicochemical conditions of the medium. Res-4′-LA is complexed by HP-β-CDs yielding a 1:1 stoichiometry complex, whereas Res-4′-DHA forms 1:2 complexes.

The performed computational simulations help us to complete the picture of the encapsulation and self-assembly equilibria associated with the novel LipoResv compounds. While absolute MMGBSA binding energies indicate stronger interactions for the LipoResv with HP-β-CDs, LE values reveal that smaller and more compact molecules, e.g., LA and DHA, lead to more efficiency on a per-atom basis, in agreement with complexation constants observed in the experiments. Furthermore, molecular dynamics simulations confirmed the ability of this complex ternary system to maintain the equilibrium between monomer LipoResv, self-assembling micelles and LipoResv-CDs complexes. Overall, the experimental results combined with multiscale modelling approaches underscore the relevance of this strategy in guiding the rational design of CDs-based encapsulation systems for poorly water-soluble bioactive phenolic compounds, such as LipoResv, with potential applications in the pharmaceutical, nutraceutical, and food science fields. This integrated experimental–computational approach not only rationalizes the encapsulation behaviour observed for LipoResv compounds but also provides a versatile framework that can be readily adapted to other amphiphilic systems exhibiting micelle formation, enabling the prediction of self-assembly processes under varying environmental conditions.

### Limitations and Future Outlook

The limitations associated with the use of HP-β-CDs for encapsulating LipoResv are partly due to the specific interaction behaviours of CDs with each compound targeted for encapsulation. Additionally, regulatory restrictions governing their use—whether in food, pharmaceutical, or environmental applications—also play a crucial role in determining their suitability.

On the other hand, the encapsulation of LipoResv using CDs is constrained by the intrinsic characteristics of lipophenols. In particular, compounds such as Resv-4′-DHA exhibit extremely low water solubility, which hinders the analysis of their behaviour—even when employing advanced micellar system-based measurement techniques.

Future research in the field of CDs is increasingly oriented toward the encapsulation of additional LipoResv derivatives from various fatty acids. The aim is to elucidate potential behavioural patterns associated with the specific fatty acid esterifying the resveratrol molecule. Furthermore, ongoing studies are exploring alternative encapsulation strategies to enhance the bioavailability of LipoResv.

## Figures and Tables

**Table 1:** CMC, complexation constant (Kc), particle size (nm), and polydispersity index (PDI) of Resv-4′-LA and Resv-4′-DHA in the presence of HP-β-CDs.

LipoResv	Concentration (µM)	HP-β-CDs (mM)	Size (nm)	%RSD	PDI	CMC (µM)	K_C_ (M^−1^)
Resv-4′-LA	10	0 10	438 89	2.64 5.08	0.42 0.57	6	719
	50	0 10	622 894	0.17 1.13	0.21 0.37		
Resv-4′-DHA	10	0 10	600 943	1.76 7.75	0.31 0.46	0.001	K_1_: 17; K_2_: 0.18
	50	0 10	2989 3835	3.18 10.37	0.73 0.95		

**Table 2:** CMC and Kc of Resv-4′-LA and Resv-4′-DHA at different temperatures (35, 25, and 15 °C), pHs (7.0 and 2.0), and ionic strengths (PBS 100 mM and MilliQ water).

LipoResv	Temperature (°C)	pH	Medium	CMC (µM)	Kc (M^−1^)
Resv-4′-LA	35	7.0	PBS	6.00	720
	25	7.0	PBS	0.27	8157
	15	7.0	PBS	0.14	10,432
	35	2.0	PBS	0.54	4347
	35	7.0	MilliQ	0.11	42,535
Resv-4′-DHA	35	7.0	PBS	1 × 10^−3^	K_1_: 17; K_2_: 0.18
	25	7.0	PBS	6 × 10^−4^	K_1_: 787; K_2_: 0.10
	15	7.0	PBS	3 × 10^−4^	K_1_: 898; K_2_: 0.33
	35	2.0	PBS	5 × 10^−4^	K_1_: 97; K_2_: 0.45
	35	7.0	MilliQ	5 × 10^−4^	K_1_: 707; K_2_: 0.33

## Data Availability

The original contributions presented in this study are included in the article/supplementary material. Further inquiries can be directed to the corresponding authors.

## Supplementary Materials

The following supporting information can be downloaded at: https://www.mdpi.com/article/10.3390/ijms26157454/s1.
