# Supplementary material for: Physicochemical and Computational Study of the Encapsulation of Resv-4′-LA and Resv-4′-DHA Lipophenols by Natural and HP-β-CDs
Source: Int J Mol Sci. 2025 Aug 1;26(15):7454. doi: 10.3390/ijms26157454 (PMC12347287; doi:10.3390/ijms26157454)
Supplement: Supplementary file 1 [file ijms-26-07454-s001.zip › ijms-3769943-supplementary.pdf]

Supplementary Materials for Physicochemical and Computational Study of the Encapsulation of Resv-4′-LA and Resv-4′-DHA Lipophenols by Natural and HP-β-CDs

Ana Belén Hernández-Heredia [1], Dennis Alexander Silva-Cullishpuma [1], José Pedro Cerón-Carrasco [2],
Ángel Gil-Izquierdo [3], Jordan Lehoux [4], Léo Faion [4], Céline Crauste [4], Thierry Durand [4],
José Antonio Gabaldón [1,*] and Estrella Núñez-Delicado [1,*]

[1] Molecular Recognition and Encapsulation Research Group (REM), Health Sciences Department,
Universidad Católica de Murcia (UCAM), Campus de los Jerónimos 135, E-30107 Guadalupe, Spain;
abhernandez@ucam.edu (A.B.H.-H.); dasilva@ucam.edu (D.A.S.-C.)

[2] Centro Universitario de la Defensa, Universidad Politécnica de Cartagena, C/Coronel López Peña s/n, Base Aérea de San Javier, E-30720 Santiago de la Ribera, Murcia, Spain; jose.ceron@cud.upct.es

[3] Research Group on Quality, Safety, and Bioactivity of Plant-Derived Foods, Department of Food Science and Technology, Centro de Edafologia y Biologia Aplicada del Segura-Consejo Superior de Investigaciones Científicas (CEBAS-CSIC,) Campus de Espinardo-25, E-30100 Murcia, Spain; angelgil@cebas.csic.es (A.G.-I.)

[4] Institut des Biomolécules Max Mousseron (IBMM), Pôle Chimie Balard Recherche, UMR 5247-CNRS,
Faculty of Pharmacy, Université de Montpellier-École Nationale Supérieure de Chimie de Montpellier (ENSCM), 34000 Montpellier, France;
jordan.lehoux@umontpellier.fr (J.L.); leo.faion@umontpellier.fr (L.F.);
celine.crauste@umontpellier.fr (C.C.); thierry.durand@umontpellier.fr (T.D.)

* Correspondence: jagabaldon@ucam.edu (J.A.G.); enunez@ucam.edu (E.N.-D.); Tel.: +34-968-278869 (E.N.-D.)

Table S1. Binding Affinities and Ligand Efficiencies for LipoResv with CDs. Best MMGBSA ΔG Bind (kcal/mol)

Molecule	α-CD	β-CD	γ-CD	Methyl-β-CD	HP-β-CD
DPHT	-43.46	-36.02	-34.48	-37.40	-47.38
Resv	-43.89	-40.70	-40.02	-38.94	-45.52
LA	-43.93	-36.90	-43.01	-38.11	-47.24
DHA	-46.14	-45.37	-47.55	-50.06	-51.05
Resv-4'-LA	-47.76	-51.40	-56.82	-50.45	-64.36
Resv-4'-DHA	-42.23	-50.80	-62.00	-53.98	-58.34

Table S2. Best Ligand Efficiency (kcal/mol per heavy atom)

Molecule	α-CD	β-CD	γ-CD	Methyl-β-CD
DPHT	-2.13	-1.85	-1.69	-1.93
Resv	-2.27	-2.20	-2.10	-2.14
LA	-1.80	-1.62	-1.67	-1.73
DHA	-1.41	-1.60	-1.28	-1.70
Resv-4'-LA	-0.88	-1.00	-0.94	-1.10
Resv-4'-DHA	-0.74	-0.80	-0.77	-0.91

Table S3. Summary of Experimental Conditions for Solubilization and Complexation Studies.

<table>
<tr><th>Compound</th><th>Concentration range (μM)</th><th>[HP-β-CDs] (mM)</th><th>pH</th><th>Temperature (°C)</th><th>Medium</th></tr>
<tr><td>LA
DHA</td><td>0, 5, 7.5, 10, 25, 50, 75, 100, 150, 200, 250, 300, 350, 400, 450, 500, 600, 700, 800, 900, 1000, 1500, 2000</td><td>0, 0.25, 0.5, 0.75, 1*</td><td>7</td><td>35</td><td>PBS</td></tr>
<tr><td rowspan="4">Resv-4′-LA
Resv-4-DHA</td><td rowspan="4">0, 0.1, 0.25, 0.5, 1, 2, 5, 7.5, 10, 20, 30, 40, 50, 60, 70, 80, 90, 100, 125, 150, 175, 200, 250, 300</td><td rowspan="4">0, 0.25, 0.5, 0.75, 1, 2, 5, 7, 10</td><td rowspan="3">7</td><td>15</td><td>PBS</td></tr>
<tr><td>25</td><td>PBS</td></tr>
<tr><td>35</td><td>PBS & MilliQ</td></tr>
<tr><td>2</td><td>35</td><td>PBS</td></tr>
</table>

* Above this concentration of CDs, no increase in fluorescence was observed, indicating that encapsulation equilibrium had been reached.

Supplementary Protocol S1. Detailed Procedure for Fluorescence Assays.

The samples were prepared in 15 mL glass test tubes with ground gocket following the procedure below:

1. Preparation of stock solutions for lipophilic compounds:
   The stock solutions of LA, DHA, Resv-4'-LA and Resv-4'-DHA were prepared in 98% ethanol at concentrations 100 times higher than the desired final concentration. For example, for a final concentration of 300 μM, a stock solution of 30 mM was prepared in ethanol.

2. Preparation of the fluorescent probe:
   Solution of 8.9 mM DPHT solution was prepared in tetrahydrofuran (THF) and stored at -80 °C. Daily, a 1:10 dilutions were carried out in PBS to use in each assay.

3. Preparation of the buffer solutions:
   Different buffers were prepared supplemented or not with the HP-β-CDs concentration necessary according to the experimental conditions:
   - PBS (100 mM, pH 7.0)
   - Milli-Q ultrapure water (18 MΩ·cm, pH 7.0)
   - Sodium borate buffer (100 mM) at pH 2.0

4. Sample preparation:
   Each sample was prepared in duplicate and contained:
   - 20 μL of the corresponding fatty acid or LipoResv stock solution
   - 1.96 mL of the previously prepared buffer solution

   The mixtures were homogenized by vortexing for 2–5 s.

   - After that, 20 μL of DPHT solution (0.89 μM final concentration in each sample) were added to the tube.

   The mixtures were homogeneized again by vortexing (2-5 s) under dark conditions and tubes were briefly purged with N_2 to remove dissolved oxygen. They were then incubated in darkness at the corresponding temperatures (15, 25 or 35 °C).

5. Experimental controls:
   - Negative control: A blank sample containing all components, but without fatty acid or LipoResv (only with 20 μL of ethanol), was incubated under identical conditions to correct signals attributable to the solvent.
   - Positive control: Solution of 40 mM Triton X-100 with 0.89 μM DPHT was incubated under identical conditions to ensure the fluorescent activity of the probe and the reproducibility of the equipment.

6. Fluorescence measurement:
   After 60 min incubation, 200 μL of each sample, positive and negative controls, were pipetted in duplicate into a black 96-well plate.

Considering that two tubes were prepared for each sample and that each tube was measured twice, the total number of measurements per sample was 4.
Fluorescence was measured at an emission wavelength of 430 nm (excitation wavelength: 358 nm) using a SpectraMax iD3 plate reader, equipped with temperature control set to the corresponding experimental condition.

7. <u>Assay repeatability:</u>
All experiments were conducted under both intraday and interday conditions to assess the reproducibility of the results, ensure proper instrument performance, and confirm the robustness of the analytical methodology. The corresponding quality control results were presented in Table S4.

Table S4. Fluorescence intensity (FI) measured intraday and interday. Negative control, some experimental samples with different concentrations of HP-β-CDs and Resv-4′-LA, and positive control at pH 7, 35 °C and PBS.

Condition	[HP-β-CDs] (mM)	[Resv-4′-LA] (μM)	Intraday FI	Intraday FI average	%RSD	Interday FI	Interday FI average	%RSD
Negative control (Blank)	0.5	-	5.51E+05	5.77E+05	6.26%	5.83E+05	5.86E+05	0.60%
			6.02E+05			5.88E+05		
	5		5.95E+06	6.02E+06	1.64%	6.09E+06	6.28E+06	4.28%
			6.09E+06			6.47E+06		
	7		9.30E+06	9.50E+06	2.90%	9.30E+06	9.14E+06	2.55%
			9.69E+06			8.97E+06		
Resv-4′-LA	0	20	2.23E+06	2.21E+06	1.60%	2.23E+06	2.53E+06	16.52%
			2.18E+06			2.82E+06		
		30	3.03E+06	2.82E+06	10.80%	3.03E+06	3.13E+06	4.52%
			2.60E+06			3.23E+06		
		60	5.09E+06	5.02E+06	2.11%	4.94E+06	4.81E+06	3.82%
			4.94E+06			4.68E+06		
	5	20	6.87E+06	6.93E+06	1.12%	6.64E+06	6.76E+06	2.41%
			6.98E+06			6.87E+06		
		30	7.54E+06	7.65E+06	1.94%	7.53E+06	7.64E+06	2.04%
			7.75E+06			7.75E+06		
		60	1.02E+07	1.03E+07	1.37%	1.09E+07	1.06E+07	4.69%
			1.04E+07			1.02E+07		
Positive control (Tritón x-100)	-	-	9.67E+07	9.89E+07	3.08%	1.01E+08	1.04E+08	3.42%
			1.01E+08			1.06E+08		
			1.03E+08	1.10E+08	8.39%	1.16E+08	1.14E+08	2.48%
			1.16E+08			1.12E+08		
			1.07E+08	1.08E+08	1.31%	1.20E+08	1.14E+08	8.10%
			1.09E+08			1.07E+08		

Notes: Fluorescence intensity (FI) was measured at 358/430 nm (excitation/emission) and is reported in arbitrary units (a.u.), as provided directly by the instrument software.

Supplementary Note S1. Detailed Fitting Procedure for Kc Determination.

The apparent binding constants (Kc) between HP-β-CDs and LipoResv or fatty acid were estimated by monitoring the variation in fluorescence intensity of the guest compound upon incremental addition of CDs. The fitting procedure was adapted from the method described by López-Nicolás et al.

As reflected in the Materials and Methods section, in subsection 3.4. determination of complexation stoichiometry and equilibrium constant between CDs and fatty acid or LipoResv, the Kc are defined by Equations 2 and 3 of the main manuscript, referred to here as S1 and S2:

$$K_1 = \frac{[LipoResv - CDs]}{[LipoResv][CDs]} \quad \text{(S1)}$$

$$K_2 = \frac{[LipoResv - CD_2]}{[LipoResv - CDs][CDs]} \quad \text{(S2)}$$

Reorganizing Equations S1 and S2, we have:

$$[LipoResv - CDs] = K_1[LipoResv][CDs] \quad \text{(S3)}$$

$$[LipoResv - CD_2] = K_1K_2[LipoResv][CDs]^2 \quad \text{(S4)}$$

If we know that the final concentration of LipoResv is defined by the following equation:

$$[LipoResv] = [LipoResv]_f + [LipoResv - CD] + [LipoResv - CD_2] \quad \text{(S5)}$$

where $[LipoResv]_f$ corresponds to free LipoResv (CMC_0), and the apparent CMC of an amphiphilic compound in the presence of CDs corresponds to the sum of the free (CMC_0) plus the complexed compound. Substituting equations S3 and S4 into S5, the following equations were obtained:

$$CMC = CMC_0 + K_1CMC_0[CDs] + K_1K_2CMC_0[CDs]^2 \quad \text{(S6)}$$

which corresponds to Equation 4 in the original manuscript, and where:

$$CMC = \frac{-(1 + K_1CMC_0) + \sqrt{[1 + K_1CMC_0)^2 + 4K_1K_2CMC_0CD_t]}}{2\,K_1K_2CMC_0} \quad \text{(S7)}$$

Non-linear regression fitting was performed by using a GraphPad Prims 9 to analyse.
